# Climatic effects on mosquito abundance in Mediterranean wetlands

**DOI:** 10.1186/1756-3305-7-333

**Published:** 2014-07-16

**Authors:** David Roiz, Santiago Ruiz, Ramón Soriguer, Jordi Figuerola

**Affiliations:** 1 Wetland Ecology Department, Doñana Biological Station, CSIC, Sevilla, Spain; 2 Present address: UMR 224 MIVEGEC/BEES, IRD, Montpellier, France; 3 Diputación de Huelva, Área de Medio Ambiente, Servicio de Control de Mosquitos, Huelva, Spain

**Keywords:** Mosquitoes, Climate change, Temperature, Rainfall, GAM, GLM, West Nile, *Plasmodium*, Dirofilariasis

## Abstract

**Background:**

The impact of climate change on vector-borne diseases is highly controversial. One of the principal points of debate is whether or not climate influences mosquito abundance, a key factor in disease transmission.

**Methods:**

To test this hypothesis, we analysed ten years of data (2003–2012) from biweekly surveys to assess inter-annual and seasonal relationships between the abundance of seven mosquito species known to be pathogen vectors (West Nile virus, Usutu virus, dirofilariasis and *Plasmodium* sp.) and several climatic variables in two wetlands in SW Spain.

**Results:**

Within-season abundance patterns were related to climatic variables (i.e. temperature, rainfall, tide heights, relative humidity and photoperiod) that varied according to the mosquito species in question. Rainfall during winter months was positively related to *Culex pipiens* and *Ochlerotatus detritus* annual abundances. Annual maximum temperatures were non-linearly related to annual *Cx. pipiens* abundance*,* while annual mean temperatures were positively related to annual *Ochlerotatus caspius* abundance. Finally, we modelled shifts in mosquito abundances using the A2 and B2 temperature and rainfall climate change scenarios for the period 2011–2100. While *Oc. caspius*, an important anthropophilic species, may increase in abundance, no changes are expected for *Cx. pipiens* or the salt-marsh mosquito *Oc. detritus*.

**Conclusions:**

Our results highlight that the effects of climate are species-specific, place-specific and non-linear and that linear approaches will therefore overestimate the effect of climate change on mosquito abundances at high temperatures. Climate warming does not necessarily lead to an increase in mosquito abundance in natural Mediterranean wetlands and will affect, above all, species such as *Oc. caspius* whose numbers are not closely linked to rainfall and are influenced, rather, by local tidal patterns and temperatures. The final impact of changes in vector abundance on disease frequency will depend on the direct and indirect effects of climate and other parameters related to pathogen amplification and spillover on humans and other vertebrates.

## Background

Vector-borne diseases (VBD) represent a serious threat to human health [1] and wildlife conservation [2]. Over the last three decades and concurrent to the emergence of newly recognized diseases, the incidence and geographic distribution of VBD have increased dramatically [3] and now account for almost one third of all cases of emerging diseases [4]. Although VBD transmission is determined by many factors, from host immunity to pathogen circulation, in recent years great emphasis has been placed on the notion that climate change may alter patterns of VBD transmission [5-10]. Climate influences various aspects of the vector-pathogen-host system and the existence of a close relationship between climate and the force of infection (i.e. the rate at which a population of hosts is infected) has been hypothesized [11]. Currently, our understanding of the underlying mechanisms that influence mosquito-borne disease transmission cycles is not complete and the potential impact of global warming on these diseases is today a subject of on-going debate and a source of controversy [7,12-15].

Climate has direct effects on vectors (e.g. abundance, distribution and longevity), pathogens (e.g. incubation period, replication and lineage), hosts (e.g. abundance, distribution and behaviour) and their interactions. Mosquito abundance is an important factor that conditions vectorial capacity and R_o_ (the basic reproductive rate), and high abundance is often a prelude to an epidemic [7,10]. Previous studies have concluded that high temperatures and high rainfall are positively related to mosquito abundance; even so, in some cases mosquito abundance peaks are known to be preceded by periods of drought (Additional file 1).

West Nile virus (WNV), Chikungunya, Dengue and Usutu are considered to be (re)-emerging in Europe [9,16-18]. However, only limited information regarding the influence of climatic variables on the population dynamics of vectors in Europe is available [19-21]. In particular, WNV has become widespread [22-25] and disease outbreaks have occurred when epizootic vectors transmit this virus from birds to horses or humans [26]. Wetlands are linked to endemic WNV circulation [27] and here we study two wetlands in SW Spain where: 1) WNV circulation since at least 2003 has been documented regularly in resident birds [28,29], horses [30] and humans [31]; 2) there is a great abundance of several mosquito species potentially involved in disease transmission [32,33] (Table 1); and 3) outbreaks of WNV in humans and horses have been reported from a neighbouring province [34-36]. In addition, the mosquito-borne flavivirus Bagaza has been detected in the surrounding area [37], there is a high incidence of *Dirofilaria immitis* and the potential malarial vector *Anopheles atroparvus* is abundant (Table 1).

**Table 1:** **Larval habitats and role in pathogen transmission of the main mosquito species detected in Doñana**[31,32,37,40-42,44,65]

**Mosquito species**	**Larval habitats**	**Vectorial competence (laboratory)**	**Field detection in Doñana**	**Role as WNV vector**
***Culex pipiens***	Inundation areas	West Nile virus	West Nile virus	Enzootic and epizootic West Nile vector
	Rice fields	Rift Valley virus		
	Man-made water bodies.			
	Nearly every kind of water source.			
***Culex perexiguus (univittatus)***	Swamps	West Nile virus	West Nile virus	Enzootic and epizootic West Nile vector
	Ponds		Usutu virus	
	Ground pools			
	Water wells.			
***Culex modestus***	Irrigation channels	West Nile virus		Enzootic West Nile virus vector
	Rice fields	Tahyna		
	Ground pools	Tularemia		
	Ponds			
	Marshes.			
***Culex theileri***	Marshes	*Dirofilaria immitis*, West Nile virus		
	Ditches	Rift Valley virus Sindbis		
	Swamps			
	Rice fields			
***Ochlerotatus caspius***	Halophilic species	West Nile virus	Mosquito Marsh virus	
	Salt marshes, Tidal coastal marshes	Tahyna Tularaemia		
***Ochlerotatus detritus***	Halophilic species			
	Salt marshes, Tidal coastal marshes			
***Anopheles atroparvus***	Canals	Malaria		
	Ditches			
	Marshes			
	Rice fields			

Consequently, we investigated the relationships between several climatic variables and the female abundance of seven mosquito species on inter-annual and seasonal scales in the period 2003–2012. We also modelled the potential effects of climate change on mosquito abundance using downscaled General Circulation Model outputs under two of the emission scenarios postulated by the Spanish National Meteorological Agency (AEMET).

## Methods

### Study area

**Figure 1:**
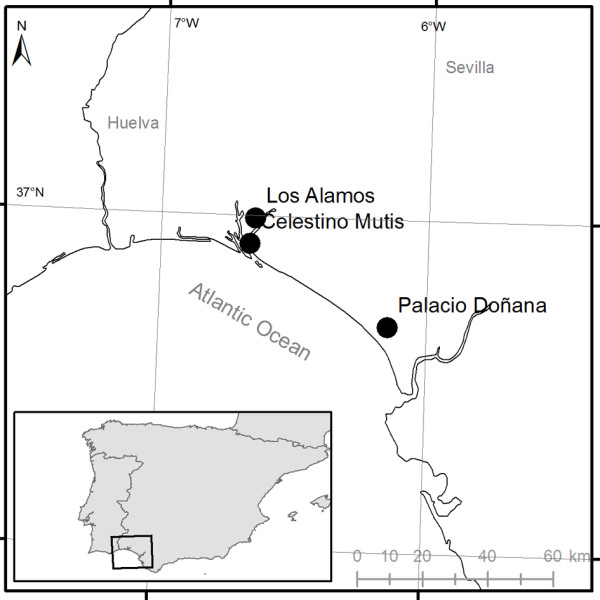
Map of the study area.

The studied locations lie within the Doñana National Park and Odiel Natural Park in SW Spain (Figure 1). These two sites are included on the Ramsar list of wetlands of international importance and are classified as Biosphere reserves by UNESCO. The climate is Mediterranean subtropical in type, with hot dry summers and frequent rainfall in autumn and winter. The mean, maximum and minimum temperatures reported are 18.3, 31.6 and 7.7°C, respectively, while mean annual rainfall is 516 mm (maximum monthly mean rainfall of 83 mm in December and minimum 1 mm in June). Doñana is a strongly seasonal freshwater wetland, while Odiel is very influenced by tides and has brackish wetlands. The most representative plants in the seasonal freshwater coastal areas are *Scirpus maritimus* L.*, S. littoralis* Schrader*, Typha latifolia* L., and *Phragmites australis* (Cav.) Trin., while in tidal salt-marshes the vegetation is mainly composed of halophyte species such as *Spartina densiflora* Brongn, *Salicornia ramossisima* J. Woods, *Sarcocornia perennis* (Miller) A. J. Scott and *Arthrocnemum macrostachyum* (Moric.). In the Odiel salt-marshes operational mosquito control with the larvicide *Bacillus thuringiensis* var. *israeliensis* (Bti) (VectoBac 12 AS, Valent Biosciences, Libertyville, IL, USA) is carried out on a fortnightly basis every year from March to October. The avian communities of these wetlands are characterized by both migratory and sedentary species of ducks, herons, gulls, waders and other birds, with over 400 species and several millions of individuals recorded. Wild and domestic mammals (horses and cows) are also common, mainly in Doñana, and more than four million people visit the area annually, with a daily influx of more than 10,000 tourists during spring and summer.

### Mosquito sampling and data

CDC (Centre for Disease Control) traps with 6-volt batteries were supplemented with a dry-ice tank of 1 kg capacity. Traps were located 2 m above ground level and were operated in 24-hour cycles. Trapping took place twice a week in February–November in 2003–2012. Traps were placed at three locations in areas of natural habitat for mosquitoes: Los Álamos (hereafter Álamos), Celestino Mutis (hereafter Mutis) and Palacio de Doñana (hereafter Palacio). Alamos and Mutis are near the tidal marshes of the Tinto and Odiel rivers, whilst Palacio is surrounded by the freshwater marshes of the Guadalquivir River (Figure 1). All three areas are environmentally protected and no landscape changes occurred in the area surrounding the traps during the study period. Mosquitoes were killed and transported to the laboratory on dry ice. Mosquitoes were counted, sexed and identified using taxonomic keys [38,39] with a stereomicroscope and a chill table.

### Environmental variables

**Figure 2:**
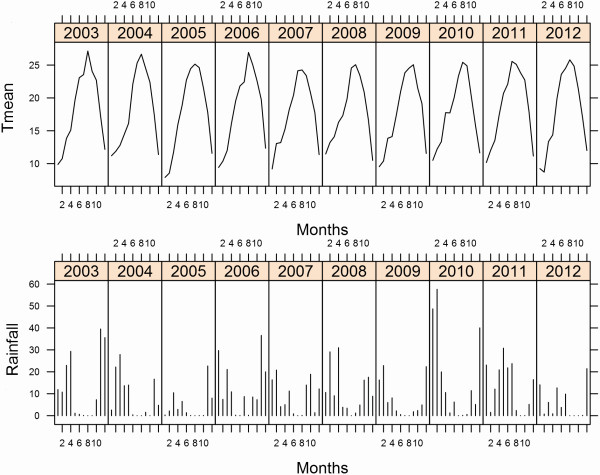
Seasonal dynamics of temperature and rainfall in the period 2003–2012.

Temperature (mean, maximum, minimum), rainfall, relative humidity (mean, maximum and minimum), wind speed and direction, solar radiation, evapotranspiration and daylight hours during the study period were extracted from data from the closest meteorological stations to the trapping localities: Las Torres, Tomejil Moguer (http://www.mapa.es/siar/Informacion.asp) and Palacio de Doñana (http://www-rbd.ebd.csic.es/mediofisico/parametrosmeteorologicos/palaciomanual/em05.htm). Tidal data were obtained from the *Anuarios de Mareas del Instituto Hidrográfico de la Marina* for the ports of Mazagón (Huelva) and Bonanza (Cadiz) at the mouth of the Guadalquivir River. Data were pooled by week and the mean weekly values were computed for all variables except for rainfall, for which the total weekly precipitation was used (Figure 2).

### Statistical analysis of intra-annual (seasonal) patterns

To control for temporal autocorrelation, we fitted an autoregressive time series model to the weekly female mosquito abundance data for each mosquito species, selecting the complexity with a model selection procedure based on Akaike’s Information Criterion (AIC). With the order of the autoregressive time series, we fitted an ARIMA model to each female mosquito abundance time series. We extracted the residuals of the ARIMA that were used as the response variable for the intra-annual models. The explanatory variables were mean, maximum and minimum temperature, tide height, mean and total rainfall, wind speed, wind direction, solar radiation, evapotranspiration and photoperiod (daylight hours). Given that a time lag operates for the effects of climatic variables on larval abundance (and therefore on adult mosquito abundance), it is important to use aggregated climatic variables to detect biological relationships [40]. Therefore, a series of accumulated temperature, rainfall and tide variables were calculated for 1–4 weeks before the sampling week. The variables used in the analysis were the accumulated average temperature, accumulated rainfall and accumulated tide of 1–2, 1–3, 1–4, 2–3, 2–4 and 3–4 weeks before the sampling week. When the explanatory variables are highly correlated to each other, backward selection can select suboptimal models due to collinearity [41]. To select a set of unrelated independent variables, we compared the Variance Inflation Factor (VIF) of covariates, then excluded the covariate with the highest VIFs, recalculated the correlation and repeated the process until all VIF values were lower than four [42]. Based on this exploratory analysis, we selected the following explanatory variables: mean temperature during the week of capture, accumulated temperatures 1–4 weeks before capture, the accumulated tide heights two weeks before capture, accumulated tide heights 2–3 weeks before capture, mean relative humidity, wind speed, sum of rainfall during the week of capture, accumulated rainfall one week before capture, and accumulated rainfall 1–2 and 2–4 weeks before capture. Accumulated tide variables were only analysed in relation to the salt-marsh mosquitoes *Ochlerotatus caspius* and *Oc. detritus,* two species that use larval habitats that depend on tidal patterns. Given that the photoperiod is an important variable with a close collinearity to temperature, we performed a GLMM analysis for all species that only included photoperiod. For *Cx. theileri*, *Cx. modestus* and *An. atroparvus* we only used data from Palacio since in the two other localities these species were absent or only present in very low numbers. To model relationships between female mosquito abundance (residuals of the temporal autocorrelation model) and climatic covariates we performed a GLMM with Gaussian error. We fitted the models by pseudo-likelihood [43] and the minimal models were chosen using AICs for each variables and a forward stepwise model selection procedure [44]. All the independent variables were log10-transformed. Statistical analyses were performed using the R statistical package version 2.13.1 (2011 The R Foundation for Statistical computing).

### Statistical analysis of inter-annual patterns

The effects of environmental variables on the estimates of annual average female mosquito abundances (2003–2012) in the commonest captured species were analyzed using Generalized Additive Models (GAMs). Given that the count data presented a significant overdispersion, the model was fitted using a negative binomial distributed error and a logarithm link [45]. The average number of female mosquitoes was used as the response variable for each of the seven commonest species. The explanatory variables were as follows: average annual maximum temperature, annual mean temperature, average annual minimum temperature, annual rainfall and winter rainfall. Winter rainfall was calculated as the accumulated rainfall between September (week 40) and March of the following year (week 10). These variables were modelled including ‘locality’ as an independent factor and assuming that mean values for abundance may differ spatially. Model selection was performed using AICs for each variable and using a forward stepwise model selection procedure [44]; validation was performed based on Zuur [46]. The inter-annual analysis was only performed for *Cx. pipiens, Oc. caspius* and *Oc. detritus* because these mosquito species were the only ones regularly captured in all three localities. Statistical analyses were performed with the R statistical package version 2.13.1 (2011 The R Foundation for Statistical computing) using packages mgcv, lattice and MuMin, among others.

### Climate change scenarios

The Spanish National Meteorological Agency (AEMET) has developed climate projections for the whole of the twenty-first century (http://www.aemet.es/es/elclima/cambio_climat/escenarios). We decided to use the ‘Analogue INM’ method used by the AEMET given that this statistical downscaling method, which presents the estimated data for temperature and rainfall in area E009 (Doñana) in the high-resolution reference grid for peninsular Spain and the Balearic Islands, provides the finest spatial resolution. This method provided us with the expected temperature (minimum and maximum) and rainfall values for the Doñana area using two General Circulation Models (GCMs), ECHAM (Max Planck Institute for Meteorology, Germany) and CGCM (Canadian Centre for Climate Modelling and Analysis), under the A2 and B2 emission scenarios. Scenario A2 is more extreme, and assumes self-reliance, the preservation of local identities, moderate economic development and a high growth in the global population; although energy consumption is high and changes in land use are moderately high, resources become scarce and technological change is fragmented but slower than under other scenarios. On the other hand, scenario B2 is more moderate than A2 and emphasizes environmental preservation and social equity with local solutions for economic, social and environmental sustainability; the global population is expected to increase continuously, yet more slowly than under scenario A2. B2 allows for a moderate level of economic development (like A2), but with lower energy consumption and fewer changes in land use; resources are more abundant and technological change is more diverse than under A2.

Based on the inter-annual models, we projected inter-annual variation in modelled female mosquito abundance based on the AEMET modelling and calculated the percentage shift in projected female mosquito abundance in the period 2011–2100 in relation to the reference period 1961–1990. Analyses were carried out using R software.

## Results

### Mosquito sampling

**Figure 3:**
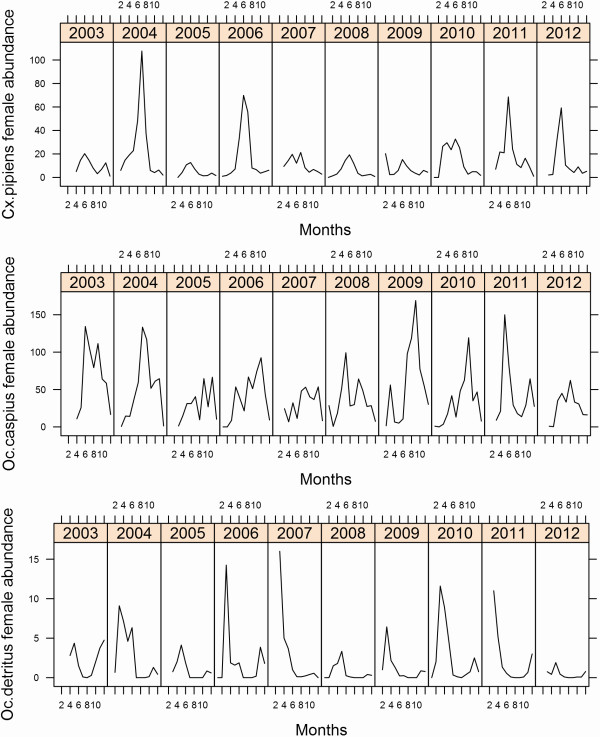
Seasonal dynamics of the three commonest mosquito species for all localities in the period 2003–2012.

**Figure 4:**
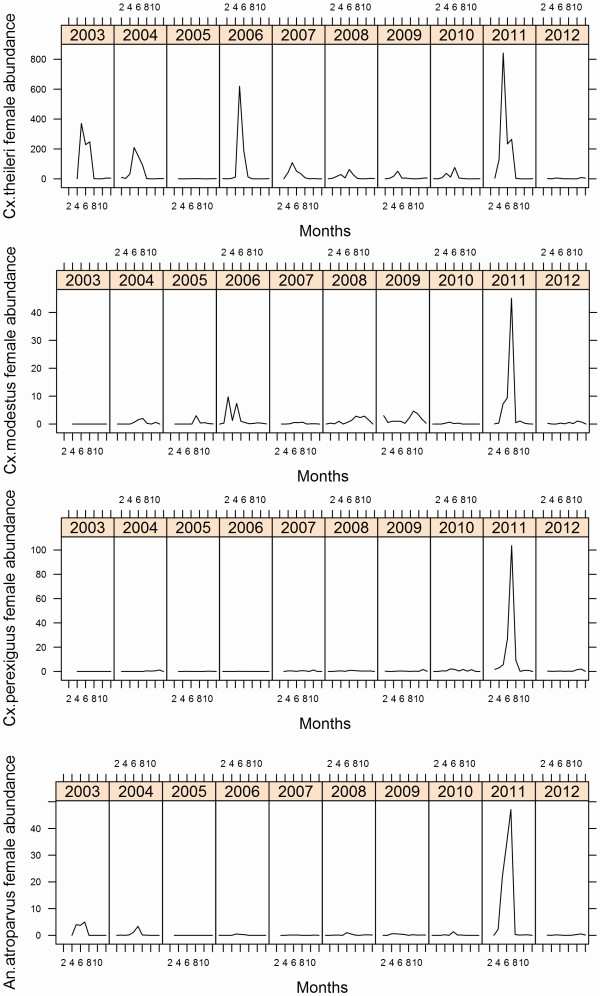
Seasonal dynamics of the four mosquito species present only at Palacio during the period 2003–2012.

In total, 184 326 female mosquitoes belonging to seven species were captured (Table 2): *Anopheles atroparvus* Van Thiel*, Culex pipiens* Linnaeus*, Culex theileri* Theobald*, Culex modestus* Ficalbi*, Culex perexiguus* Theobald*, Ochlerotatus caspius* (Pallas) and *Ochlerotatus detritus* (Haliday). *Oc. caspius* was the most abundant species throughout the whole season, despite displaying a certain seasonality in its cycles (Figure 3). *Cx. pipiens* and *Oc. detritus* were abundant in all three localities in spring–summer and spring–autumn, respectively. *Cx. theileri, Cx. modestus, Cx. perexiguus* and *An. atroparvus* were especially abundant at Palacio (Table 2 and Figure 4).

**Table 2:** Number of female mosquitoes captured for the seven more abundant species collected between 2003–2012 in the three studied localities

**Locality**	***Oc. caspius *** **(%)**	***Oc. detritus *** **(%)**	***Cx. pipiens *** **(%)**	***Cx. theileri *** **(%)**	***Cx. modestus *** **(%)**	***Cx.perexiguus *** **(%)**	***An.atroparvus *** **(%)**	**Total mosquitoes**
Álamos	47015 (81.46)	1093 (1.89)	9159 (15.84)	324 (0.56)	105 (0.18)	18 (0.03)	1 (0.01)	57715
Mutis	13468 (60.32)	1100 (4.93)	7065 (31.64)	414 (1.85)	170 (0.76)	98 (0.44)	11 (0.05)	22326
Palacio	42902 (41.14)	544 (0.52)	1592 (1.53)	56680 (54.35)	1883 (1.81)	319 (0.31)	365 (0.35)	104285
Total	103385 (56.09)	2737 (1.48)	17816 (9.67)	57418 (31.15)	2158 (1.17)	435 (0.24)	377 (0.2)	184326

### Intra-annual relationships of climate and female mosquito abundance

Mean temperature was positively related to *Cx. pipiens* and *Oc. caspius* abundances (Table 3). Accumulated temperatures in the period 1–4 weeks (one month) before capture were positively related to *Cx. perexiguus* and *Cx. modestus,* and negatively to *Cx. pipiens* and *Oc. detritus* abundances. Accumulated rainfall during the period 2–4 weeks before capture was positively related to *Oc. detritus* abundance. Accumulated tide heights for two weeks before capture were positively related to *Oc. caspius* abundance and accumulated tide heights during 2–3 weeks before capture to *Oc. detritus* abundance. Mean relative humidity was negatively related to *Cx. theileri* and *An. atroparvus* abundances (Table 3). None of the other variables were significant (Additional file 2). The photoperiod (hours of light) was positively related to *Cx. perexiguus, Cx. theileri, An. atroparvus* and *Oc. caspius* abundances but was negatively related to *Oc. detritus* abundance (Table 4).

**Table 3:** Results of the models (GLMM gaussian) for the significant climatic variables related to the seasonal patterns (intra-annual) of female abundance for seven species of mosquitoes

**Dependent variable**	**Independent variables**	**Coefficient (±S.E)**	**t-value**	**df**	**p-value**
***Culex pipiens***	Intercept	5.5012 ± 3.6666			
	Mean temperature	0.9404 ± 0.3449	2.726	1,787	0.0065
	Accumulated temperature 1–4 weeks	-0.3240 ± 0.0910	-3.560	1, 787	0.0004
***Culex perexiguus***	Intercept	-1.4462 ± 0.4680			
	Accumulated temperature 1–4 weeks	0.0213 ± 0.0061	3.473	1, 214	0.0006
***Culex modestus***	Intercept	-3.7820 ± 1.2065			
	Accumulated temperature 1–4 weeks	0.0624 ± 0.0158	3.946	1, 214	0.0001
***Culex theileri***	Intercept	90.7469 ± 44.2425			
	Mean relative humidiy	-1.3653 ± 0.6473	-2.109	1, 214	0.0361
***Anopheles atroparvus***	Intercept	2.1637 ± 0.6365			
	Mean relative humidity	-0.0337 ± 0.0093	-3.623	1, 214	0.0003
***Ochlerotatus caspius***	Intercept	-306.1215 ± 75.4695			
	Accumulated tide 2 weeks before	6.34085 ± 1.8209	3.482	1, 787	0.0005
	Mean temperature	1.9980 ± 0.6247	3.198	1, 787	0.0014
***Ochlerotatus detritus***	Intercept	-9.2316 ± 2.4513			
	Accumulated tide 2–3 weeks before	0.2489 ± 0.0586	4.241	1, 786	<0.0001
	Accumulated temperature 1–4 weeks	-0.0260 ± 0.0111	-2.343	1, 786	0.0194
	Accumulated rainfall 2 to 4 weeks	0.02949 ± 3.3909	0.006	1, 786	<0.0001

**Table 4:** Results of the models (GLMM gaussian) for the relationship between photoperiod (hours of light) and the seasonal patterns (intra-annual) of female abundance for seven species of mosquitoes

**Dependent variable**	**Independent variables**	**Coefficient (±S.E)**	**F**	**df**	**p-value**
***Culex pipiens***	Intercept	-10.48657 ± 6.391346			
	Photoperiod	18.44723 ± 10.870908	1.697	1, 788	0.0901
***Culex modestus***	Intercept	0.02061 ± 2.44786			
	Photoperiod	1.52955 ± 4.66071	0.328	1, 214	0.743
***Culex perexiguus***	Intercept	-2.1168 ± 0.9235			
	Photoperiod	4.2972 ± 1.7547	2.449	1, 214	0.0151
***Culex theileri***	Intercept	-173.79 ± 52.79			
	Photoperiod	331.21 ± 100.51	3.295	1, 214	0.00115
***Ochlerotatus caspius***	Intercept	-85.99189 ± 26.18172			
	Photoperiod	157.28911 ± 45.26570	3.475	1, 788	0.0005
***Ochlerotatus detritus***	Intercept	3.026271 ± 1.499752			
	Photoperiod	-6.133728 ± 2.703791	2.269	1, 788	0.0236
***Anopheles atroparvus***	Intercept	-2.3264 ± 0.7793			
	Photoperiod	4.2522 ± 1.4838	2.866	1, 214	0.00458

### Interannual relationship of climatic variables and female mosquito abundance

**Figure 5:**
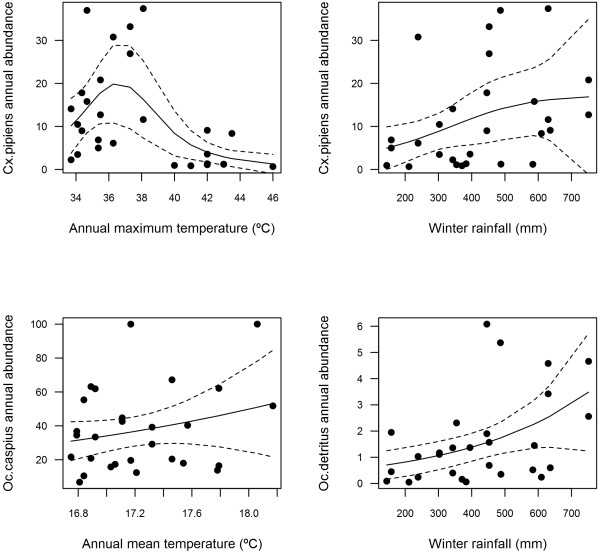
Relationship (GAMs) of climatic variables and interannual patterns of mosquito abundance.

*Cx. pipiens* abundance was positively related to annual maximum temperatures and winter rainfall. Annual mean temperatures were positively related to *Oc. caspius* (Table 5; Figure 5), while *Oc. detritus* abundance was positively related to winter rainfall.

**Table 5:** Results of the models (Generalized Additive Models) for the significant climatic variables related to inter-annual (between years) patterns of female abundance for three species of mosquitoes that were captured regularly in the three localities

**Dependent variable**	**Independent variables**	**df**	**Chi-sq**	**p-value**	**Deviance explained (%)**
***Culex pipiens***	Annual maximum temperature	2	7.694	0.02134	72.8
	Winter rainfall	2	11.467	0.00324	
***Ochlerotatus caspius***	Annual mean temperatures	1.73	11.61	0.00293	55.7
***Ochlerotatus detritus***	Winter rainfall	1	6.656	0.00988	43.7

### Climate change scenarios

**Figure 6:**
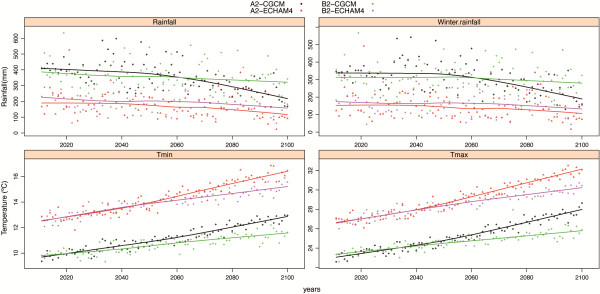
Projected temperature and rainfall for the period 2011–2100 according to climate change scenarios A2 and B2 and GCMs ECHAM4 and CGCM.

**Figure 7:**
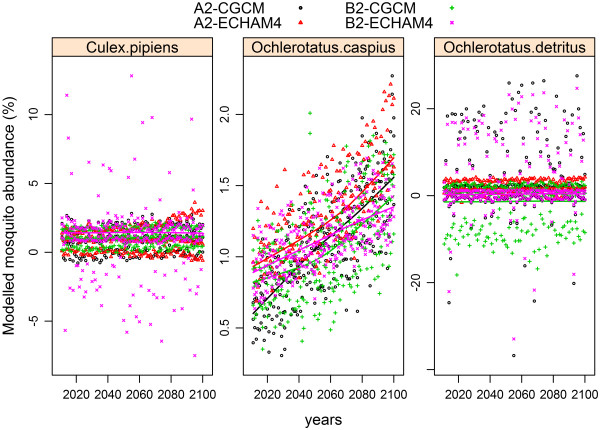
Percentage of change of female mosquito abundance in the period 2011–2100 in relation to the reference period 1961–1990 according to the relationships between temperature and rainfall and mosquito abundance reported in our study and the climatic projections for scenarios A2 and B2.

Using the increase in temperatures and decrease in rainfall in the study area forecasted by the scenarios (Figure 6), we projected variations in the abundances of female *Cx. pipiens, Oc. caspius* and *Oc. detritus*. The other mosquito species were not represented because their inter-annual analysis did not give any significant variable. Under climate change scenarios A2 and B2 for the period 2013–2100, it is predicted that *Oc. caspius* abundance will considerably increase but that *Oc. detritus* and *Cx. pipiens* abundances will not change (Figure 7). Although projections do not change qualitatively between scenarios, larger quantitative changes in mosquito populations are expected under the A2 scenarios.

## Discussion

Mosquito abundance is related to climatic variables, although, depending on the species in question and the temporal scale used, the key variables will vary. Temperatures in general were positively related to mosquito abundance and can affect survival rates, mortality, larval productivity and other population parameters [7,10]. Weekly temperatures were related to seasonal abundance patterns in *Cx. pipiens* and *Oc. caspius*, while accumulated temperatures (1–4 weeks before) were positively related to *Cx. modestus* and *Cx. perexiguus* abundances. These results are consistent with previous studies in USA, South Africa and Russia that link high summer temperatures to great vector abundance and WNV outbreaks [20,47-49]. On the other hand, accumulated temperatures (1–4 weeks before) were negatively related to *Cx. pipiens* and *Oc. detritus* abundances. These negative relationships, like the negative effect of annual maximum temperatures over 40°C on *Cx. pipiens* abundance (Figure 5), may reflect the negative effect of high temperatures on mosquito longevity and life expectancy [50]. These two mosquito species may be more sensitive to high temperatures, although an indirect effect may also exist given that high evapotranspiration and the absence of larval habitats coincide with the period of highest temperatures.

Accumulated rainfall (2–4 weeks before) is positively related to *Oc. detritus* weekly abundance. We did not detect any relationship between drought periods and mosquito abundance as has been reported in other studies [51-53]. However, both *Cx. theileri* and *An. atroparvus* within-year abundances are negatively related to relative humidity, which could be due to drought, to an effect on flight activity, or to high evapotranspiration. In general, the relationship rainfall-mosquito abundance is variable and depends on differences in the ecology and habitat selection patterns of each mosquito species.

The influence of tide heights on the abundance of salt-marsh species has previously been reported by [54]: both *Oc. caspius* and *Oc. detritus* weekly abundances were related to tide height, albeit with a time lag of 1–2 weeks before capture in the former and of 2–3 weeks in the latter. This lag corresponds to the fortnightly seasonal rhythms of these species, which benefit from the high tides that flood the saltmarshes and create larval breeding sites [38]. We did not detect any effect of wind on within-year abundance patterns. Wind may negatively affect captures by reducing flight activity of mosquitoes [55] and it is important to remember that the number of captures in mosquito traps depends not only on mosquito abundance but also on mosquito activity [56].

Photoperiod (day-length) was positively related to the within-year abundance patterns of *Cx. perexiguus, Cx. theileri, Oc. caspius* and *An. atroparvus*, but negatively to *Oc. detritus.* Day-length is an important variable in spring emergence and autumn decline, especially in species that enter into diapause, and probably affects the seasonal dynamics of mosquito abundance in several species. Unlike temperature or rainfall, the seasonal pattern in day-length does not vary from year to year and provides a highly reliable anticipatory cue for future seasonal conditions [57]. However, few studies have ever considered the photoperiod as an important variable affecting seasonal dynamics in mosquito populations [58]. Overall, our results agree with several previous studies (Additional file 1) that conclude that higher temperatures and greater accumulated temperatures, as well previous (winter) rainfall, are all important factors that help explain mosquito abundance.

Annual temperatures were related non-linearly to the inter-annual patterns of mosquito abundance in *Cx. pipiens* (maximum temperature) and *Oc. caspius* (mean temperatures). The influence of annual rainfall on mosquito abundance is a controversial question (Additional file 1); the results of our study show that previous rainfall is an important variable that is positively related to *Cx. pipiens* and *Oc. detritus* annual abundances.

When analysing long-term series of mosquito abundance data it is important to consider the effects that changes in larvicide treatments may have had. In our case it is unlikely that our results were affected by changes in pesticide treatment given that no such treatment is used within 45 km of Palacio. At the other two sites, however, the tidal areas suitable for *Oc. caspius* and *Oc. detritus* larval development were treated with larvicides, but at a distance of over 1 km from the traps. The product and amount used did not change over time and treatment was applied in a fortnightly cycle after high tides. This periodicity would have hindered rather than favoured our ability to detect any relationship between tides and population size within seasons and probably had no effect on inter-annual analysis or climate change modelling. Additionally, no landscape changes that could have affected the mosquito populations were detected during the study period.

The Spanish National Meteorological Agency AEMET projections suggest that climate change will have particularly marked effects in southern Spain and will lead to higher temperatures and lower rainfall [59]. Given the projections under A2 and B2 climate change scenarios and assuming the existence of a causal relationship between the climatic variables we studied and female mosquito abundance, we do not expect that any important shift (increase or decrease) in either *Cx. pipiens* (a WNV and USUV vector) or the salt-marsh mosquito *Oc. detritus* abundances will occur*.* The already abundant and anthropophilic pest mosquito *Oc. caspius,* a potential vector of diseases such as Tahyna, WNV and tularemia [38], could greatly increase in abundance, especially under the A2 climate change scenario (Figure 7), that is, more extreme estimates of change than under B2. *Oc. caspius* has a great resistance to high temperatures and drought, is active, bites at high temperatures (up to 36°C in adults and 33°C in larvae), and tolerates high salinities due to its great capacity for osmotic regulation [60]. This species depends heavily on areas flooded with brackish water in estuaries during high tides in the Atlantic and, consequently, its populations are less rainfall dependent than other species. In Doñana, we have described its bloodmeal behaviour as mammalophilic, since more than 90% of its blood meals are derived from mammals, but it has little importance in the amplification of WNV in the area [32]. In addition, in recent years the novel flavivirus Marisma Mosquito Virus has been detected in *Oc. caspius* pools analysed in the area [61], although the potential pathogenicity (if any) of this virus for vertebrates has yet to be demonstrated. We expect that the nuisance caused by *Oc. caspius* to local populations will increase in the future, although its implications for disease dynamics are unclear. *Oc. detritus* and *Oc. caspius* depend on tide patterns that vary in terms of sea levels and so, hypothetically, rising sea levels under global warming could lead to an increase in salt-marsh mosquito abundance [62]. Projections of seasonal changes in rainfall patterns suggest a decrease in spring and summer precipitations and an increase in the length of drought periods [63]. Consequently, we would also expect changes in the phenology of mosquito abundance. The reliability of these projections depends on the nature of the relationship between climate and mosquito abundance, which will have to be confirmed with manipulative experiments in addition to the observational evidence presented here since the relationship between climate and life-history processes are not likely to be linear [71]. The next challenge is to accurately understand the contribution of multiple interacting and often non-linear underlying responses in hosts, pathogens and vectors to climate [64].

## Conclusions

When we reviewed the studies analysing correlations between climatic variables and WNV human cases and infection rates, the same variables (temperature and rainfall) were related to vector mosquito abundance, infection rates and cases of infection in humans (Additional file 1). Moreover, mosquito abundance has been directly related to the basic reproductive rate R_o_ (the total number of secondary cases arising from one infective case in a susceptible population) and the vectorial capacity C (the daily rate at which secondary cases arise from a currently infective case) [7,10]. Additionally, temperature alters the replication of the virus in the mosquito – the extrinsic incubation period (EID) – and therefore infection rates [48,65]. In this work, we focus on how climate change could influence transmission by altering mosquito vector abundance. An important question, given that models for vector-borne diseases use the number of vectors per host, is whether the number of vectors caught in a trap is related to the number of vectors per host [66]. Come what may, the effects of temperature are complex due to the fact that the climate-mosquito abundance relationship is species-specific and time-dependent, and long data series are needed to continue the study of these trends in mosquito abundance. Our results highlight the fact that these effects are non-linear (see Figure 5, *Cx. pipiens* and maximum temperatures) and that, therefore, linear approaches will overestimate the effect of climate change on mosquito abundances at high temperatures. Without additional studies our results are not generalizable to other habitats since relationships may vary between areas and may be place-specific [49,51]. Although it is likely that anthropogenic changes in modified wetlands will be more determinant than climate [67], this study was conducted in natural wetlands unaffected by any serious anthropogenic change such as population increase or agricultural and urban expansion. Thus, our results show that climate change does not necessarily lead to an increase in mosquito populations. Simplistic and generalist statements assuming that higher temperatures lead to more mosquitoes are not adequate and it is essential to carry out a careful analysis of temporal patterns in field data. In addition, climate is likely to affect other important parameters for disease transmission (such as vector survival and pathogen development rates) and, consequently, it is not possible to extrapolate from conclusions regarding climate and mosquito abundances to the risk of disease outbreaks. Other non-climatic variables (i.e. socio-economic development, circulation of pathogenic strains, pathogen replication, host ecology, anthropogenic changes, land use/land-cover and inundation patterns) may also be determinant when analysing the risk of vector-borne disease emergence [68]. Greater understanding of the ecology of vector-borne disease is essential if we are to understand the effects of global change on vector-borne disease outbreaks, an important challenge for future decades.

## Competing interests

The authors declare that they have no competing interests.

## Authors’ contributions

SR designed and participated in field sampling; DR, SR and JF organised the analyses, results and discussion; DR analysed the data; JF and RS provided financial support. All the authors were involved in the drafting of the manuscript and gave their approval to the final version.

## Supplementary Material

Additional file 1 Review of several studies of the effect of climatic variables on WNV cases, mosquito infection rate and vector abundance. Click here for file

Additional file 2 **Results of the models (GLMM gaussian) for all the climatic variables that are related with seasonal patterns (intra-annual) of female abundance for seven mosquito species and the estimates of the final model selected by backward selection. Significant variables are in bold. **The significance (F, p) of the non-significant variables corresponds to the final model plus the non-significant variable. Click here for file
