# Supplementary material for: Climatic effects on mosquito abundance in Mediterranean wetlands
Source: Parasit Vectors. 2014 Jul 16;7:333. doi: 10.1186/1756-3305-7-333 (PMC4223583; doi:10.1186/1756-3305-7-333)
Supplement: Additional file 1 — Review of several studies of the effect of climatic variables on WNV cases, mosquito infection rate and vector abundance. [file 1756-3305-7-333-S1.doc]

**Additional file 1:** Review of several studies of the effect of climatic variables on WNV cases, mosquito infection rate and vector abundance.

| **Dependent variable** | **Independent climatic variables** | **Mosquito vector species** | **Geographical area** | **References** |
| --- | --- | --- | --- | --- |
| WNV human cases | Annual rainfall from previous year |  | USA | [Landesman et al., 2007](#_ENREF_28), |
|  | Increasing temperature | *Cx. pipiens,*  *Cx. perexiguus* | Israel | Paz and Albersheim, 2008 |
|  | Increasing temperature  Relative humidity | *Cx. pipiens,*  *Cx. perexiguus* | Israel | Paz, 2006 |
|  | Accumulated temperature |  | USA | [Soverow et al., 2009](#_ENREF_47) |
|  | Previous drought,  coindidence wetting | *Cx. nigripalpus* | Florida | Shaman et al., 2002,  2005, |
|  | Drought |  | Romania | Savage et al., 1999 |
|  | Mean temperature | *Cx. pipiens,*  *Cx. modestus* | Russia | Platonov et al., 2008 |
| WNV infection rates | Previous and annual rainfall | *Cx. univitattus* | South Africa | [Uejio et al., 2011](#_ENREF_49) |
|  | Increasing temperature  Drought | *Culex sp.*  *(pipiens-restuans*  *and salinarius)* | NY, USA | [Shaman et al., 2011](#_ENREF_43) |
|  | Increasing temperature  Decreasing rainfall | *Cx. pipiens*  *Cx.restuans* | USA | Ruiz et al., 2010 |
| Vector abundance | Previous rainfall  Accumulated temperature | *Culex sp.*  *(pipiens-restuans*  *and salinarius)* | New Jersey,  USA | DeGaetano, 2005 |
|  | Increasing temperature | *Cx. pipiens,*  *Cx tarsalis* | Seattle, USA | Pecoraro et al., 2007 |
|  | Increasing temperature  Rainfall | *Cx.modestus,*  *Cx. pipiens,*  *Oc. caspius* | Camargue,  France | Poncon et al., 2007 |
|  | Increasing temperature  Previous rainfall | *Cx.tarsalis*  *Cx. restuans* | Dakota,  USA | Chuang et al., 2011 |
|  | Temperature, water | *Cx. pipiens-restuans* | NY, USA | Trawinski & MacKay 2008 |
|  | Previous rainfall  Increasing temperatures | *Cx. tarsalis* | California,  USA | Reisen et al., 2008 |
|  | Accumulated precipitation  Drought | *Ae. vexans*  *Cx.nigripalpus* | SW Georgia,  USA | Buckner et al., 2011 |
|  | Degree days | *Cx.pipiens,*  *Cx. restuans* | Illinois,  USA | Kunkel et al., 2006 |
|  | Previous high tide, temperature and  rainfall | *Ae. sollicitans* | Northeast  USA | Shone et al., 2006 |
|  | Accumulated temperature,  Previous rainfall | *Cx.pipiens-restuans* | Ontario,  Canada | Wang et al., 2011 |
