# Supplementary material for: Climatic effects on mosquito abundance in Mediterranean wetlands
Source: Parasit Vectors. 2014 Jul 16;7:333. doi: 10.1186/1756-3305-7-333 (PMC4223583; doi:10.1186/1756-3305-7-333)
Supplement: Additional file 2 — Results of the models (GLMM gaussian) for all the climatic variables that are related with seasonal patterns (intra-annual) of female abundance for seven mosquito species and the estimates of the final model selected by backward selection. Significant variables are in bold. The significance (F, p) of the non-significant variables corresponds to the final model plus the non-significant variable. [file 1756-3305-7-333-S2.doc]

**Additional file 2:** Results of the models (GLMM gaussian) for all the climatic variables that are related with seasonal patterns (intra-annual) of female abundance for seven mosquito species and the estimates of the final model selected by backward selection. Significant variables are in bold. The significance (F, p) of the non-significant variables corresponds to the final model plus the non-significant variable.

| **Dependent variable** | **Independent variables** | **Coefficient (±S.E)** | **F** | **df** | **p-value** |
| --- | --- | --- | --- | --- | --- |
| ***Culex pipiens*** | **Intercept** | **5.5012±3.6666** |  |  |  |
|  | **Accumulated temperature 1–4 weeks** | **-0.3240±0.0910** | **-3.5596** | **1, 787** | **0.0004** |
|  | **Mean temperature** | **0.9404±0.3449** | **2.7262** | **1,787** | **0.0065** |
|  | Mean relative humidity |  | -0.0369 | 1,785 | 0.9705 |
|  | Wind speed |  | 0.4571 | 1,785 | 0.6477 |
|  | Weekly rainfall |  | -0.6392 | 1,785 | 0.5228 |
|  | Accumulated rainfall 1 week before |  | 1.6968 | 1,785 | 0.0901 |
|  | Accumulated rainfall 1 to 2 weeks |  | 0.2555 | 1,785 | 0.7984 |
|  | Accumulated rainfall 2 to 4 weeks |  | 0.2177 | 1,785 | 0.8277 |
| ***Culex modestus*** | **Intercept** | **-3.78207±1.20654** |  |  |  |
|  | **Accumulated temperature 1–4 weeks** | **0.06246±0.01583** | **3.946** | **1, 214** | **0.0001** |
|  | Mean temperature |  | -0.697 | 1,213 | 0.4864 |
|  | Mean relative humidity |  | -1.088 | 1,213 | 0.2778 |
|  | Wind speed |  | 0.002 | 1,213 | 0.99808 |
|  | Weekly rainfall |  | -0.246 | 1,213 | 0.805749 |
|  | Accumulated rainfall 1 week before |  | 1.077 | 1,213 | 0.28273 |
|  | Accumulated rainfall 1 to 2 weeks |  | -0.008 | 1,213 | 0.993250 |
|  | Accumulated rainfall 2 to 4 weeks |  | -0.543 | 1,213 | 0.587745 |
| ***Culex perexiguus*** | **Intercept** | **-1.446207±0.468089** |  |  |  |
|  | **Accumulated temperature 1–4 weeks** | **0.021331±0.006141** | **3.473** | **1, 214** | **0.0006** |
|  | Mean temperature |  | 0.516 | 1, 214 | 0.6065 |
|  | Mean relative humidity |  | -0.740 | 1,213 | 0.4603 |
|  | Wind speed |  | 0.044 | 1,213 | 0.9647 |
|  | Weekly rainfall |  | -1.409 | 1,213 | 0.1602 |
|  | Accumulated rainfall 1 week before |  | -0.005 | 1,213 | 0.9960 |
|  | Accumulated rainfall 1 to 2 weeks |  | 0.341 | 1,213 | 0.7337 |
|  | Accumulated rainfall 2 to 4 weeks |  | 0.255 | 1,213 | 0.7992 |
| ***Culex theileri*** | **Intercept** | **90.7469±44.2425** |  |  |  |
|  | **Mean relative humidiy** | **-1.3653±0.6473** | **-2.109** | **1, 214** | **0.0361** |
|  | Mean temperature |  | 0.572 | 1,213 | 0.5680 |
|  | Accumulated temperature 1–4 weeks |  | 0.037 | 1, 213 | 0.9700 |
|  | Wind speed |  | 0.404 | 1,213 | 0.6869 |
|  | Weekly rainfall |  | 1.19 | 1,213 | 0.2763 |
|  | Accumulated rainfall 1 week before |  | -0.709 | 1,213 | 0.4793 |
|  | Accumulated rainfall 1 to 2 weeks |  | -1.254 | 1,213 | 0.2111 |
|  | Accumulated rainfall 2 to 4 weeks |  | -1.007 | 1,213 | 0.3150 |
| **Dependent variable** | **Independent variables** | **Coefficient (±S.E)** | **F** | **df** | **p-value** |
| ***Ochlerotatus caspius*** | **Intercept** | **-306.1215±75.46959** |  |  |  |
|  | **Accumulated tide 2 weeks before** | **6.34085±1.82096** | **3.482155** | **1, 787** | **0.0005** |
|  | **Mean temperature** | **1.99803±0.62470** | **3.198360** | **1, 787** | **0.0014** |
|  | Accumulated tide 2-3 weeks before |  | 0.487309 | 1, 786 | 0.6262 |
|  | Accumulated temperature 1–4 weeks |  | 0.167497 | 1, 786 | 0.8670 |
|  | Mean relative humidity |  | -1.180338 | 1, 786 | 0.2382 |
|  | Wind speed |  | -0.483433 | 1, 786 | 0.6289 |
|  | Weekly rainfall |  | -1.620175 | 1, 786 | 0.1056 |
|  | Accumulated rainfall 1 week before |  | 0.151099 | 1, 786 | 0.8799 |
|  | Accumulated rainfall 1 to 2 weeks |  | 0.252289 | 1, 786 | 0.8009 |
|  | Accumulated rainfall 2 to 4 weeks |  | 0.585997 | 1, 786 | 0.5580 |
| ***Ochlerotatus detritus*** | **Intercept** | **-9.231686±2.4513901** |  |  |  |
|  | **Accumulated tide 2-3 weeks before** | **0.248927±0.0586959** | **4.240966** | **1,786** | **<0.00001** |
|  | **Accumulated temperature 1–4 weeks** | **-0.026025±0.0111083** | **-2.342880** | **1,786** | **0.0194** |
|  | **Accumulated rainfall 2 to 4 weeks** | **0.029493±3.3909** | **0.0063365** | **1,786** | **<0.00001** |
|  | Accumulated tide 2 weeks before |  | 1.822764 | 1, 785 | 0.0687 |
|  | Wind speed |  | 1.145932 | 1, 785 | 0.2522 |
|  | Average weekly rainfall |  | -1.383592 | 1, 785 | 0.1669 |
|  | Accumulated rainfall 1 week before |  | -1.41363 | 1, 785 | 0.1579 |
|  | Accumulated rainfall 1 to 2 weeks |  | -0.085829 | 1, 785 | 0.9316 |
| ***Anopheles atroparvus*** | **Intercept** | **2.163771±0.636577** |  |  |  |
|  | **Mean relative humidity** | **-0.033742±0.009314** | **-3.623** | **1, 214** | **0.000365** |
|  | Mean temperature |  | -0.303 | 1, 213 | 0.76197 |
|  | Accumulated temperature 1–4 weeks |  | -0.556 | 1, 213 | 0.57913 |
|  | Wind |  | 0.191 | 1, 213 | 0.84852 |
|  | Weekly rainfall |  | -0.411 | 1, 213 | 0.68140 |
|  | Accumulated rainfall 1 week before |  | 0.112 | 1, 213 | 0.910622 |
|  | Accumulated rainfall 1 to 2 weeks |  | -0.489 | 1, 213 | 0.62570 |
|  | Accumulated rainfall 2 to 4 weeks |  | 0.349 | 1, 213 | 0.727161 |
